# Parkinson's Disease, Speech and Neurosurgery

**DOI:** 10.1002/brb3.70101

**Published:** 2025-04-29

**Authors:** Thomas Ollivier, Serge Pinto, Anne‐Sophie Rolland, Emeline Cailliau, Gustavo Touzet, S. Thobois, A. Eusebio, E. Hainque, T. Rouaud, S. Drapier, D. Guehl, D. Maltete, M. Anheim, O. Lagha Boukbiza, C. Giordana, M. Tir, L. Hopes, C. Hubsch, B. Jarraya, A. Marques, C. Brefel, O. Rascol, J. C. Corvol, I. Benatru, Luc Defebvre, David Devos, Caroline Moreau, For the French PREDISTIM study group

**Affiliations:** ^1^ CHU Lille Department of Medical Pharmacology University of Lille, Lille Neuroscience and Cognition INSERM, U1172, LiCEND, NS‐Park/F‐CRIN Network Lille France; ^2^ Neurology and Movement Disorders Department Expert Center for Parkinson's Disease, Lille University Medical Center Lille France; ^3^ CNRS, LPL, Aix‐Marseille Université Aix‐en‐Provence France; ^4^ Biostatistics Department CHU Lille Lille France; ^5^ Neurosurgical Department Lille University Medical Center Lille France; ^6^ Faculté de Médecine Lyon Sud Charles Mérieux; CNRS, Institut des Sciences Cognitives, Univ Lyon, Université Claude Bernard Lyon 1 Bron France; ^7^ Center Expert Parkinson Hôpital Neurologique “Pierre Wertheimer”, Hospices Civils de Lyon, NS‐PARK/FCRIN Network Lyon France; ^8^ Service de Neurologie et Pathologie du Mouvement and UMR CNRS AP‐HM, Hôpital de La Timone, Aix‐Marseille Université Aix‐en‐Provence France; ^9^ Département de Neurologie Hôpital Pitié‐Salpêtrière, AP‐HP, Faculté de Médecine de Sorbonne Université, UMR S 1127, INSERM U 1127; CNRS UMR 7225; and Institut du Cerveau et de la Moëlle Epinière, NS‐PARK/FCRIN Network Paris France; ^10^ Department of Neurology Nantes University Hospital, Nantes Cedex, NS‐Park/FCRIN Network Nantes France; ^11^ Department of Neurology Rennes University Hospital, CIC INSERM 1414, NS‐PARK/FCRIN Network Rennes Cedex France; ^12^ CHU de Bordeaux, Center Expert Parkinson, Institut des Maladies Neuro‐Dégénératives Bordeaux France; ^13^ Department of Neurology Rouen University Hospital and University of Rouen Rouen France; ^14^ Laboratory of Neuronal and Neuroendocrine Differentiation and Communication INSERM U1239, NS‐PARK/FCRIN Network Mont‐Saint‐Aignan France; ^15^ Service de Neurologie Hôpitaux Universitaires de Strasbourg Strasbourg France; ^16^ Institut de Génétique et de Biologie Moléculaire et Cellulaire (IGBMC) INSERM‐U964/CNRS‐UMR7104/Université de Strasbourg Illkirch France; ^17^ Fédération de Médecine Translationnelle de Strasbourg (FMTS) Université de Strasbourg, NS‐PARK/FCRIN Network Strasbourg France; ^18^ Neurology Department Center Hospitalier Universitaire de Nice, Université Côté d'Azur Nice France; ^19^ Department of Neurology, Department of Neurosurgery, Expert Center for Parkinson's Disease Amiens University Hospital, EA 4559 Laboratoire de Neurosciences Fonctionnelles et Pathologie (LNFP) Université de Picardie Jules Verne, University of Picardy Jules Verne (UPJV), NS‐PARK/FCRIN Network Amiens France; ^20^ Neurology Department Nancy University Hospital Nancy France; ^21^ Hôpital Fondation Ophtalmologique A de Rothschild, Unité James Parkinson, NS‐Park French Network Paris France; ^22^ Pôle Neurosciences Foch Hospital Suresnes France; ^23^ Inserm Avenir, Neurospin CEA Saclay Center, Bâtiment 145, F‐91191 Gif‐sur‐Yvette France; ^24^ Neurology Department, Université Clermont Auvergne, IGCNC, Clermont‐Ferrand University Hospital NS‐PARK/FCRIN Network Clermont‐Ferrand France; ^25^ Department of Clinical Pharmacology and Neurosciences, Parkinson Expert Center, Center d'Investigation Clinique CIC1436, ToNIC UMR 1214, NeuroToul COEN Center, and NS‐PARK/FCRIN Network University Hospital of Toulouse, University of Toulouse 3, INSERM Toulouse France; ^26^ Service de Neurologie, Center Expert Parkinson, CIC‐INSERM 1402, CHU Poitiers, NS‐PARK/FCRIN Network Poitiers France

**Keywords:** Parkinson's disease, speech disorders, sub‐thalamic deep brain stimulation

## Abstract

**Background:**

Speech impairment is a recognized but unpredictable adverse effect of sub‐thalamic nucleus deep brain stimulation (STN‐DBS) for Parkinson's disease (PD).

**Objectives:**

To evaluate the prevalence of speech impairment 1 year after STN‐DBS in PD patients and to determine the predictive factors for speech outcome following STN‐DBS.

**Methods:**

Data for 417 patients from the French national PREDISTIM study were collected preoperatively. The combined effect of medical treatment and surgery on speech was compared using specific items from dedicated clinical scales (MDS‐UPDRS III.1: primary endpoint) and patient self‐assessment questionnaires (items 34 and 35 of the PDQ39: secondary endpoints). For each variable, three patient groups were generated according to speech outcome at 1 year: worsening, stability, and improvement. In the second step analysis, the three groups were compared for demographic and clinical variables at baseline and STN‐DBS parameters.

**Results:**

There was a significant deterioration in speech of all considered items 1 year after combined STN‐DBS and dopaminergic treatment. Four predictive factors for speech deterioration were detected: (i) the absence of preoperative speech impairment (*p* < 0.001); (ii) severity of motor activity of daily living (MDS‐UPDRS II off total score) (*p* = 0.037); (iii) high‐intensity stimulation of the left electrode (i.e., above 3.6 V) (*p* = 0.046); and (iv) the absence of any change in non‐motor experiences of daily life (MDS‐UPDRS I total score) (*p* = 0.048).

**Conclusions:**

Speech outcome should be carefully monitored after STN‐DBS, especially in PD patients without preoperative speech impairment, with motor difficulties in daily‐living activities, and with increased left electrode intensity.

**Trial Registration:**

ClinicalTrials.gov identifier: NCT02360683.

## Graphical abstract

This article presents the progression of speech 1 year after subthalamic neurostimulation surgery in Parkinson's disease and the predictive factors identified.

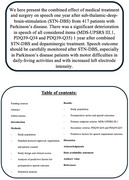

**Abbreviations**
BEST ON: with levodopa and with stimulation at V1
CGIS: clinical impression of severity
DBS: deep brain stimulation
FOGQ: freezing of gait questionnaire
HAM‐A: Hamilton scale of anxiety
LARS: Lille apathy rating scale
LEDD: levodopa equivalent daily dose
MDS‐UPDRS: Movement Disorders Society‐Unified Parkinson's Disease Rating Scale
MoCA: Montreal cognitive assessment
OFF DRUG: without levodopa at V0
OFF DRUG ON STIM: without levodopa and with stimulation at V1
ON DRUG OFF STIM: without levodopa and without stimulation at V1
ON DRUG: with levodopa at V0
PD: Parkinson's disease
PDI: polytomous discrimination index
PDQ‐39: Parkinson's disease quotation‐39
PDQ39‐Q34: question 34 of the PDQ‐39 scale
PDQ39‐Q35: question 35 of the PDQ‐39 scale
QoL: quality of life
STN‐DBS: subthalamic nucleus‐deep brain stimulation
WORST OFF: without levodopa and without stimulation at V1

## 1 Introduction

The main features of Parkinson's disease (PD) dysarthria are dysprosodia, dysphonia, hypophonia, and paroxysmal rhythmic disorders (Darley, Aronson, and Brown 1968). In PD, the impairments can concern only speech (i.e., dysarthria) or be associated with pulmonary dysfunction (i.e., dysarthropneumophonia) (Moreau et al. 2011).

Although speech disturbances are common in very advanced PD, they can occur earlier at any stage of the disease (Moreau and Pinto 2019). Eventually, dysarthria will affect about 89% of patients with PD along disease progression, affecting social interaction and contributing to poor quality of life (QoL) and depression (Muñoz‐Vigueras et al. 2021). The main issue for patients is that the impact of symptomatic drugs, notably levodopa, remains very limited on speech and is mainly limited to lip motility (Pinto et al. 2004).

Assessment of dysarthria in PD remains a challenge, and in routine evaluation, item III.1 of the Movement Disorders Society‐Unified Parkinson's Disease Rating Scale (MDS‐UPDRS) is widely used to perceptually assess the speech impairment and its progression: scores of 0 and 1 mainly refer to dysprosody and slight hypophonia, with additional slight impairment of intelligibility for a score of 2, while scores of 3 and 4 report a marked alteration of intelligibility because of more severe dysarthria (Goetz et al. 2008).

In patients with severe motor fluctuations and very high levodopa sensitivity, sub‐thalamic nucleus deep brain stimulation (STN‐DBS) is a benchmark treatment. While some speech dimensions can be improved individually following STN‐DBS, it is common to say that speech and communication worsen at some point after surgery, due to a deterioration in intelligibility and some paroxysmal events (stuttering or dystonia linked to cortico‐bulbar and/or cerebello‐cortical current diffusion) (Moreau and Pinto 2019; Gervais‐Bernard et al. 2009; Skodda et al. 2014). Predictive factors for poor outcome have been reported on a large study on speech intelligibility outcomes after DBS: on‐drug poor intelligibility before surgery, longer disease duration, medially placed left hemisphere, and left high voltage (Tripoliti et al. 2014; Wang et al. 2006). On a very limited number of patients, the threshold of 3.7 volts (V) at the left electrode has already been reported as critical for the onset of dysarthria (Törnqvist, Schalén, and Rehncrona 2005). The recent EARLYSTIM study, involving patients with an average age of 52.4 in the stimulated group, demonstrated the absence of speech degradation by studying intelligibility. On the other hand, UPDRS III item 18 showed a slight and significant deterioration in the stimulated group compared with the best medical treatment group (Pinto et al. 2023).

In this prospective, multicenter study, speech intelligibility was assessed and analyzed preoperatively and postoperatively in a large French cohort of 417 patients with PD (Boussac et al. 2020; Betrouni et al., 2022). The primary objective was to describe the impact of STN‐DBS on speech impairment after 1 year with both perceptual evaluation performed by neurologists and self‐reported questionnaires provided to the patients. The secondary objective was to confirm and refine predictive factors for the impact of STN‐DBS on speech and to define the patients at risk of speech worsening.

## 2 Methods

### 2.1 Study Population

This study was an ancillary analysis using data collected from the PREDISTIM cohort. PREDISTIM is an ongoing prospective, multicenter study sponsored by the University Hospital of Lille, conducted in 17 expert centers for PD belonging to the French clinical research network (NS‐Park/F‐CRIN). Briefly, consecutive patients undergoing STN‐DBS in each of the participating centers were included in this study, which was carried out between November 2013 and September 2019. The inclusion criteria were those of the PREDISTIM trial: a diagnosis of PD according to the UK PD Brain Bank; disease duration ≥ 5 years; age 18‒75 years; and indication for STN‐DBS. Exclusion criteria included: atypical PD; severe cognitive impairment (Montreal Cognitive Assessment (MoCA) ≤ 23); severe psychiatric disorder; acute levodopa motor response < 30%; and any medical contraindications to surgery. Clinical data were collected at baseline (V0) and 1‐year postsurgery (V1).

### 2.2 Standard Protocol Approval, Registration, and Patient Consent

This study was approved by the dedicated ethics committee (French CPP, Comité de Protection des Personnes, Nord Ouest‐IV, Protocol 2013‐A00193‐42) and was registered on the ClinicalTrials.gov website (NCT02360683). All patients gave their written informed consent before being included in the study, which was conducted according to Good Clinical Practice and local regulations. Data collection was compliant with General Data Protection Regulation rules.

### 2.3 Study Design and Clinical Scores

The primary endpoint was the speech item of the MDS‐UPDRS motor part (III.1), which is a perceptual scale ranging from 0 (no speech problems) to 4 (unintelligible speech). This criterion was assessed at baseline (V0) during two conditions of an acute test of L‐dopa challenge: (i) without dopaminergic medication (L‐dopa) for at least 12 h (OFF drug); and (ii) under L‐dopa (ON drug). This criterion was also analyzed at V1 during the same acute test of L‐dopa challenge, with and without STN‐DBS; thus, four conditions were tested: (i) with STN‐DBS and without drug (OFF drug ON stim); (ii) without STN‐DBS and without drug (WORST OFF); (iii) with levodopa but without STN‐DBS (ON drug OFF stim); and (iv) with both STN‐DBS and drug (BEST ON). As the framework of the study was to study speech in real‐life conditions, the MDS‐UPDRS III.1 score was compared under therapeutic conditions (i.e., between ON DRUG at V0 and BEST ON at V1). A qualitative distribution of the nature of speech change following surgery was considered, with three possible outcomes that would be either: (i) improvement (I: if at least 1 point lower on the score); stability (S); or worsening (W: if at least 1 more point on the score).

The secondary criterion included items 34 (PDQ39‐Q34) and 35 (PDQ39‐35) of the self‐assessment PDQ39 questionnaire, these items being respectively specific to speech (“[Over the last month], have you had difficulty with your speech?”) and communication (“[Over the last month], have you felt unable to communicate with people properly?”). These two items are discrete and quantitative and range from 0 (never) to 4 (always). The same qualitative distribution of the nature of speech change following surgery was considered for PDQ39‐Q34 and PDQ39‐Q35 items.

### 2.4 Analysis of Predictive Factors for Postoperative Speech Outcome

The following variables at V0 were studied as possible predictive factors for postoperative speech outcome: age; sex; disease duration from first symptoms and from diagnosis; serious vascular or infectious complications related to STN‐DBS; Hoehn and Yahr scale ON and OFF at V0; clinical global impression of severity (CGIS) of the disease; levodopa equivalent daily dose (LEDD); PDQ‐39 total score; MoCA; Lille Apathy Rating Scale (LARS); Hamilton scale of anxiety (HAM‐A); MDS‐UPDRS subscores (i.e., MDS‐UPDRS I total score, MDS‐UPDRS II total score ON and OFF, dopasensitivity of global motor score and of speech, MDS‐UPDRS IV total score, axial signs subscores: III.3 for neck stiffness, III.9 for chair lift, III.10 for walking, III.11 for freezing of gait, III.12 for postural stability, and III.13 for posture). Question 4 of the freezing of gait questionnaire (FOGQ) was chosen to represent the severity of gait freezing at V0. The left electrode stimulation amplitude (V) was also considered.

### 2.5 Statistical Analysis

Categorical variables (sex and MDS‐UPDRS III.1 ≤ 1/4) are described as frequency and percentage. Quantitative variables are described as mean ± standard deviation (SD) in the case of a normal distribution (Shapiro–Wilk test) or median (interquartile range, IQR) if the distribution was not normal. Baseline and postsurgery speech scores were compared using the Student's *t*‐test or Wilcoxon signed rank test in cases of non‐normal distribution. For each of MDS‐UPDRS III.1, PDQ39‐Q34, and PDQ39‐Q35 scores, patients were divided into three groups according to the changes between baseline and postsurgery scores (improvement, stability, or worsening). Potential predictive factors were compared between the three groups using the Chi (Moreau et al. 2011) test for sex, and analysis of variance (ANOVA) or Kruskal–Wallis test for quantitative variables, depending on normal or non‐Gaussian data distribution, respectively. Significant factors at *p* = 0.20 were introduced into a multivariable multinomial logistic regression model, and independent predictors were identified using a backward selection procedure.

Odds ratios (OR) and their 95% confidence intervals [95% CI] were derived from multinomial models as effect sizes, using the “stability” group as a reference. For multivariate analyses, missing values in candidate predictive factors were handled by multiple imputation procedures. Missing data were imputed under the missing at random assumption using a regression switching approach (chained equation with *m* = 10 imputations) with the predictive mean matching method for quantitative variables and logistic regression (binary, ordinal, or multinomial) for qualitative variables (van Buuren and Groothuis‐Oudshoorn, 2011). The imputation procedure was performed using all potential predictive factors and outcomes (pre‐ and postsurgery scores), and estimates obtained in the different imputed data sets were combined using Rubin's rules (Van Calster et al. 2012). The performance of the selected multivariable model was examined in terms of discrimination by calculating the Polytomous Discrimination Index (PDI) (Van Calster et al. 2012); the results are reported as median (range: min–max) values across the imputed datasets (Marshall et al. 2009). The optimal intensity threshold for the MDS‐UPDRS‐III.1 score to predict worsening (vs. stability or improvement) was determined to maximize the positive predictive value. Statistical testing was conducted at the two‐tailed *α*‐level of 0.05. Statistical analyses were performed using SAS software version 9.4 (SAS Institute, Cary, NC).

## 3 Results

### 3.1 Study Population

Among the 795 patients enrolled in the PREDISTIM cohort, 417 were included in this ancillary speech study because they had data available for the primary endpoint (MDS‐UPDRS III.1) at V0. Then data from 309 of these patients were analyzed both pre‐ and postoperatively. For PDQ39‐Q34 and PDQ39‐Q35, these analyses concerned 401 and 406 patients, respectively.

Two‐thirds (*n* = 271) of the study participants were male and the mean (± SD) age of the whole group was 60.1 ± 7.6 years. Only 12 of these patients had local hemorrhagic or infectious complications that required the replacement of material. The mean (± SD) duration of the disease was 11.4 ± 4.3 years; median Hoehn and Yahr score varied from 2 (IQR: 2‒3) to 1 (IQR: 1‒2) from OFF to ON drug at V0. Mean (± SD) LEDD was 1344 ± 564 mg. The population consisted of patients without dementia with low anxiety and apathy scores. Regarding the stimulation parameters, mean (± SD) left amplitude was 2.2 ± 0.6 V, mean (± SD) frequency was 130 ± 62.5 Hertz, and mean (± SD) pulse width was 60 ± 28.4 µs. Postsurgery change in the MDS‐UPDRS III scores is described below.

The characteristics of the study population are summarized in Table 1 and Table S1.

**TABLE 1:** Baseline patient characteristics.

Baseline (V0) patient characteristics	Value
Male/Female (% male)	271/146 (65)
Age (years)	60.1 ± 7.6
Disease duration (years)^a^	11.4 ± 4.3
L‐dopa equivalent daily dose (mg/day)^b^	1344 ± 564
MDS‐UPDRS III score	
OFF medication^c^	42.4 ± 15.3
ON medication^d^	10.8 ± 7.2
Mean intensity of left electrode^e^ (V)	2.2 ± 0.6

*Note*: Categorical variables are expressed as *n* (%) and quantitative variables are described as mean ± standard deviation.

Abbreviation: MDS‐UPDRS: Movement Disorders Society—Unified Parkinson Disease Rating Scale.

^a^ Calculated for 415 patients.

^b^ Calculated for 400 patients.

^c^ Calculated for 411 patients.

^d^ Calculated for 410 patients.

^e^ Calculated for 283 patients.

### 3.2 Postoperative Motor and Speech Outcomes

Total mean MDS‐UPDRS III motor score decreased from 42.4 ± 15.3 off medication to 10.8 ± 7.2 after taking levodopa at V0. At V1, the score was 44.5 ± 16.1 in WORST OFF, 22 ± 12.4 OFF drug ON stim, 19.1 ± 12.0 ON drug OFF stim, and 10.8 ± 7.2 in BEST ON. Thus, mean MDS‐UPDRS III total score increased at 1 year in WORST OFF from 42.4 ± 15.3 to 44.5 ± 16.1 (*p* = 0.006) (Table S1).

### 3.3 Primary Endpoint Criteria: MDS‐UPDRS III.1

The primary endpoint criterion (MDS‐UPDRS III.1) increased significantly between V0 ON drug and V1 BEST ON (*p* < 0.001). A total of 277 (90%) patients had a MDS‐UPDRS III.1 score of 0 or 1 preoperatively (non‐dysarthric patients), but this rate dropped to 253 (82%) patients at 1 year. Patients with no or mild dysarthria at baseline (with a MDS‐UPDRS III.1 score of 0 or 1 at V0 ON drug) had significantly greater worsening of speech postsurgery (*p* < 0.001) compared with patients with dysarthria (MDS‐UPDRS III.1 ≥ 2). Of the 277 non‐dysarthric patients at V0, 42 (15%) had dysarthria at V1. At 1‐year, only 15 (5%) of the patients had a severe speech disorder (i.e., score ≥ 3) (Table 2), and of these patients, only 3/15 already had severe dysarthria preoperatively (i.e., score ≥ 3). Of the 32 dysarthric patients at V0, 18 (56%) were no longer dysarthric at V1 (Table 2).

**TABLE 2:** Number (%) of patients according to speech scores and their progression at V1.

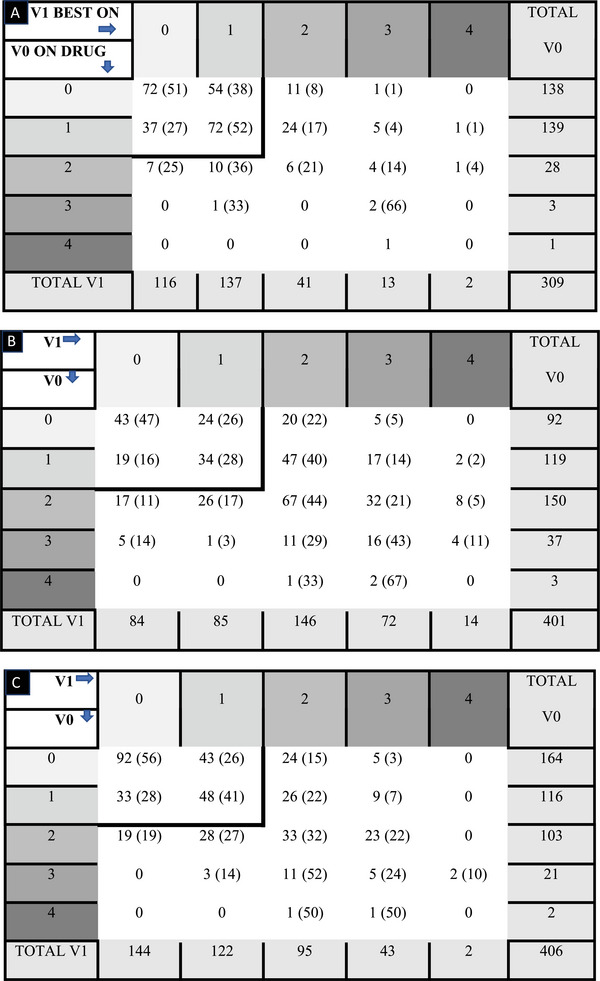

*Note*: A: score: MDS‐UPDRS III.1. V0 ON DRUG: before surgery with medication; V1 BEST ON: 1 year after surgery with both stimulation and medication. Smaller bold rectangle concerns 235 (76%) patients with a speech score of 0 or 1 at 1 year (V1). B: score: **PDQ39‐Q34**. Small bold rectangle concerns 120 (30%) patients with a speech score of 0 or 1 at 1 year (V1). C: score **PDQ39‐Q35**. Small bold rectangular concerns 216 (53%) patients with a speech score of 0 or 1 at 1 year (V1).

### 3.4 Secondary Criteria: PDQ39‐Q34 and PDQ39‐Q35

The same results were observed for items PDQ39‐Q34 and PDQ39‐Q35, with a significant increase between V0 and V1 (*p* < 0.001 and *p* = 0.010, respectively) (Table 2). It is interesting to note that for non‐dysarthric patients or those who did not experience communication disorders (i.e., those with a speech score of 0 or 1 in PDQ39‐Q34 or PDQ39‐Q35 at baseline), PDQ39‐Q34 and PDQ39‐Q35 included only 30% (120/401) of the patients and 53% of the patients (216/406) (Table 2), respectively.

Patients with no or mild dysarthria at baseline (with a PDQ39‐Q34 score of 0 or 1 at baseline) or who did not experience communication problems (with a PDQ39‐Q35 score of 0 or 1 at baseline) had significantly greater worsening of speech postsurgery (*p* < 0.001 for both) compared with patients with those problems (i.e., with PDQ39‐34 or PDQ39‐Q35 ≥ 2).

For item PDQ39‐Q34, among 277 non‐dysarthric patients at V0 (i.e., with a score at baseline of 0 or 1), 42 (15%) displayed dysarthria at V1. At 1 year, 86 patients (21%) had severe speech disorders (i.e., a score ≥ 3) (Table 2). Of these patients, 22 (26%) had previous severe dysarthria at V0 (i.e., a score ≥ 3).

For item PDQ39‐Q35, among 280 patients who did not experience communication disorders at V0 (i.e., with a score at baseline of 0 or 1), 64 (23%) experienced communication disorders at V1. At 1 year, 45 patients (11%) experienced severe communication disorders (i.e., score ≥ 3) (Table 2). Of these patients, only eight had severe communication disorders at V0 (i.e., score ≥ 3).

### 3.5 Predictive Factors for Speech Impairment Outcome

For the MDS‐UPDRS III.1 score, as well as for PDQ39‐Q35, the MDS‐UPDRS II OFF score at V0 was significantly higher in the group of patients with postoperative speech worsening (*p* = 0.032 and *p* = 0.009, respectively). In the univariate analysis, the preoperative factors significantly associated with a change in MDS‐UPDRS III.1 were: sex (the improving group containing more males than the other two groups; *p* = 0.016); disease duration (the improving group had a longer disease duration than the other two; *p* = 0.008); MDS‐UPDRS III.1 speech score of 0 or 1 (overrepresented among those stable or worsening; *p* < 0.001); preoperative total scores for MDS‐UPDRS II and III in the OFF condition (the improving group having higher scores overall; *p* = 0.032 and *p* = 0.046); and left electrode intensity (the higher, the worse; *p* < 0.001) (Table S2). The preoperative factors significantly associated with the change in PDQ39‐Q34 were preoperative anxiety (HAM‐A: the stable group was the least anxious; *p* = 0.049) and QoL (PDQ39: the improving group was the one with the highest score; *p* = 0.015) (Table S3). The factor associated with the change in PDQ39‐Q35 was the PDQ39 score (*p* = 0.031) (Table S4).

The multivariate predictive model of the change in MDS‐UPDRS III.1 score at V1 showed four predictive parameters (Table 3). Compared to speech stability, speech worsening was associated with higher left electrode stimulation intensity (OR = 1.51 [95% CI: 1.08‒2.11]), a higher MDS‐UPDRS II total OFF score at V0 (OR = 1.05 [95% CI: 1.01‒1.09]), and a lower MDS‐UPDRS I total score at V0 (OR = 0.94 [95% CI: 0.89‒1.00]). Conversely, patients (in one of the three modalities) had more chances to improve their MDS‐UPDRS III.1 score than to remain stable (OR = 8.59 [95% CI: 3.62‒20.37]). For items PDQ39‐Q34 and PDQ39‐Q35, the data from multivariate analysis are presented in Tables S5 and S6. The median (IQR) values of the PDI for the final predictive models were 0.544 (0.513‒0.569), 0.456 (0.449‒0.459), and 0.545 (0.538‒0.554) for the MDS‐UPDRS III.1, PDQ39‐Q34, and PDQ39‐Q35, respectively. The intensity threshold of 3.6 V was identified as the optimal threshold to predict speech change with a positive predictive value of 70.0%, a negative predictive value of 71.9%, a specificity of 97.9%, and a low sensitivity of 11.1% (Table 4). The population of 209 patients used to define the intensity threshold was not different from the total population (i.e., missing data for 100 patients).

**TABLE 3:** Independent predictive factors retained in the final predictive model of MDS‐UPDRS‐III.1 score change at V1.

	Improvement vs. stability		Worsening vs. stability		Overall *p* value
	OR [95% CI]	*p* value	OR [95% CI]	*p* value	
MDS‐UPDRS III.1 V0 > 1	8.59 [3.62‒20.37]	< 0.001	0.58 [0.19‒1.75]	0.33	< 0.001
MDS‐UPDRS II OFF V0	1.02 [0.97‒1.07]	0.47	1.05 [1.01‒1.09]	0.010	0.037
Left electrode intensity	1.14 [0.68‒1.91]	0.62	1.51 [1.08‒2.11]	0.016	0.046
MDS‐UPDRS I V0	0.95 [0.88‒1.02]	0.13	0.94 [0.89‒1.00]	0.026	0.048

*Note*: The median (Q1, Q3) value of the polytomous discrimination index (PDI) for the final predictive model was 0.544 (0.513‒0.569).

Abbreviation: MDS‐UPDRS: Movement Disorders Society‐Unified Parkinson Disease Rating ScalePDQ39: Parkinson's disease quality of life; V0: preoperative; V1: 1‐year postsurgery.

**TABLE 4:** Prediction thresholds of left electrode intensity (V) for speech worsening.

Threshold (V)	MDS‐UPDRS III.1 (stability or improved)	MDS‐UPDRS III.1 (worsened)	Total
< 3.6	143	56	199
≥ 3.6	3	7	10
Total	146	63	209

Abbreviations: MDS‐UPDRS: Movement Disorders Society‐Unified Parkinson Disease Rating Scale; V: volts.

## 4 Discussion

The main result of this study is that under optimal therapeutic conditions, speech worsened significantly (*p* < 0.001) 1 year after STN‐DBS in our group of PD patients. In addition, above 3.6 V at the level of the left electrode, 70% of patients showed a deterioration in their speech reflected by one additional point in the MDS‐UPDRS III.1. However, it should be noted that only 11% of patients whose speech became worse at 1 year had a left electrode intensity ≥ 3.6 V. In the absence of a patient control group, this study doesn't allow us to incriminate DBS solely, although the deterioration of the disease shouldn't be considered significant (Holden et al. 2018) (worsening of the MDS‐UPDRS III score in OFF from 42.4 to 44.5 on average between V0 and V1).

This study also quantified the differences in the assessment of speech between the clinician and the patient: preoperatively, 76% of the patients were classified as non‐dysarthric with the hetero‐assessment tool (MDS‐UPDRS III.1), while only 30% reported no impairment with the self‐assessment QoL questionnaire (PDQ39‐Q34).

The main preoperative predictive factors detected for speech deterioration at 1 year were: the absence of any speech disorder before surgery; a high motor severity score in activities of daily living in OFF before surgery (MDS‐UPDRS II OFF V0 score); high stimulation intensity at the level of the left electrode; and a low score of non‐motor experiences of daily life (MDS‐UPDRS I V0).

The population was similar to that of patients usually selected as candidates for DBS in PD (Tanaka et al. 2020; Tripoliti et al. 2014; Aviles‐Olmos et al. 2014). The low frequency of speech disorders preoperatively, with only 10% of patients with dysarthria, testifies to the homogeneity of the population selected.

The results in the literature concerning the impact of dysarthria are heterogeneous, with some reports of worsening (Tsuboi et al. 2015; Tripoliti et al. 2011; Phokaewvarangkul, Boonpang, and Bhidayasiri 2019; Hariz et al. 2008) and others of improvement (Skodda et al. 2014; Dromey et al. 2000) according to the method and nature of evaluation (acoustic, aerodynamic, perceptual…) used and the number of people concerned. Our results are consistent with worsening of speech by increasing left electrode intensity (Tripoliti et al. 2008; Törnqvist, Schalén, and Rehncrona 2005). On the other hand, our results are not in agreement with the report that the longer the duration of disease progression, the greater the deterioration of speech (Tripoliti et al. 2014). Along with initial observations, some reported that preoperative speech deterioration was a risk factor for postoperative worsening, since in our study two‐thirds of the patients who experienced speech deterioration had a score of 0 for the MDS‐UPDRS III.1. To our knowledge, no impact of pacing pulse width has been reported to date on speech (Dayal et al. 2020). On the other hand, our group and other authors have demonstrated that the use of low frequency has an influence on speech using either item III.1 of the MDS‐UPDRS or other scores in small numbers of patients (Moreau et al. 2011; Phokaewvarangkul, Boonpang, and Bhidayasiri 2019; Fabbri et al. 2019). However, such improvement was only transient and was conditioned by a deterioration in other segmental parameters such as tremor, limiting its use in the long term and reserved for the subgroup with severe preoperative dysarthria (i.e., a score for item 18 of ≥ 3) (Fabbri et al. 2019). Male gender was overrepresented in the improving group in the univariate analysis and did not predict postoperative outcome in the multivariate model. These results are in line with some published previously (Xie et al. 2011) and in contradiction with others reporting a postoperative worsening of the voice handicap index in women (Tanaka et al. 2015). We did not find any difference in the mode of speech evolution concerning either the fourth subpart of the MDS‐UPDRS score or the preoperative QoL, contrary to what was reported previously (Fabbri et al. 2019). Our study aimed to evaluate speech under “real‐life” conditions of the patients; hence, we used the BEST ON at 1 year and self‐questionnaires, independent of the ON/OFF status. The suspicion that DBS is responsible for a slight worsening of speech at 1 year may be questioned, and it should be remembered that levodopa itself can slightly worsen speech (Pinto et al. 2004), although no impact has been reported in the early stages of the disease (Tykalova et al. 2022). The results presented here measure the combined effect of stimulation and medication observed in everyday life; one of our earlier studies has already demonstrated this deleterious combined effect on speech (Pinto et al. 2014).

The strengths of this study come from the statistical power derived from the large patient number, the multicenter character, the use of a simple validated clinical tool (MDS‐UPDRS III.1), the contribution of results from many selected variables, and the use of self‐administered questionnaires that not only allow the examiner's assessment to be compared with that of the patient but also incorporate a social dimension of communication into the study through the use of question 35 of the PDQ39.

The main limitation of this study stems from the use of a single perceptual tool to assess speech, the MDS‐UPDRS III.1 subscore, which cannot account for the total complexity and heterogeneity of the dimensions of speech that can be affected. However, it has the advantage of being widely used by neurologists but is the least reproducible item (Martinez‐Martin et al. 2013). Since the examiner may not be the same between V0 and V1, it is possible that there is a bias since 1‐point variations in MDS‐UPDRS III.1 were considered. However, we tried to limit this bias of inter‐observer variability by considering dysarthric patients from 2/4, which represents the most objective threshold, after which certain words are no longer understood by the examiner. The neuroanatomical atlases used in this study do not allow for a detailed analysis of the fiber tracts involved in speech disorders, so the location of the active pad was not investigated and represents future work.

In conclusion, the absence of dysarthria pre‐STN‐DBS is not a guarantee of non‐aggravation postoperatively, and conversely, slight preoperative speech impairment is not systematically related to postoperative speech degradation. Clinicians must be attentive to predictive factors of speech outcome following surgery, paying particular attention to patients who have motor difficulties in daily‐living activities (MDS‐UPDRS II OFF V0), patients for which the intensity of stimulation at the level of the left electrode is likely to be increased, or patients who have only minor impairment in terms of non‐motor experiences (MDS‐UPDRS I V0). In the future, these predictive factors should be further confirmed and refined to better specify the expectations for speech outcome post‐STN‐DBS.

## Author Contributions

**Thomas Ollivier**: conceptualization, investigation, writing–original draft, methodology, validation, visualization, writing–review and editing, software, formal analysis, project administration, data curation, resources. **Serge Pinto**: conceptualization, methodology, writing–review and editing, visualization, validation, supervision. **Anne‐Sophie Rolland**: conceptualization, methodology, writing–review and editing, supervision, funding acquisition, validation, project administration, data curation, visualization. **Emeline Cailliau**: methodology, software, formal analysis, validation, visualization, writing–review and editing, supervision. **Gustavo Touzet**: supervision, visualization. **S. Thobois**: supervision, validation, investigation. **A. Eusebio**: investigation, validation, supervision. **E. Hainque**: investigation, validation, supervision. **T. Rouaud**: investigation, validation, supervision. **S. Drapier**: investigation, validation, supervision. **D. Guehl**: investigation, validation, supervision. **D. Maltete**: investigation, validation, supervision. **M. Anheim**: investigation, validation, supervision. **O. Lagha Boukbiza**: investigation, validation, supervision. **C. Giordana**: investigation, validation, supervision. **M. Tir**: investigation, validation, supervision. **L. Hopes**: investigation, validation, supervision. **C. Hubsch**: investigation, validation, supervision. **B. Jarraya**: investigation, validation, supervision. **A. Marques**: investigation, validation, supervision. **C. Brefel**: investigation, validation, supervision. **O. Rascol**: investigation, validation, supervision, visualization, writing–review and editing. **J.C. Corvol**: investigation, validation, supervision. **I. Benatru**: investigation, validation, supervision. **Luc Defebvre**: conceptualization, methodology, supervision, validation, writing–review and editing, visualization, funding acquisition, investigation. **David Devos**: conceptualization, methodology, validation, visualization, writing–review and editing, funding acquisition, investigation, formal analysis, project administration, supervision. **Caroline Moreau**: conceptualization, methodology, supervision, formal analysis, validation; investigation, funding acquisition, visualization, writing–review and editing, project administration.

The French PREDISTIM group members are as follows:

## – Lille

– Neurologists: Dr. Caroline Moreau, Pr. Luc Defebvre, Dr. Nicolas Carriere, Dr. Guillaume Grolez, Dr. Guillaume Baille, Dr. Kreisler
– Neuroradiologists: Pr. Jean‐Pierre Pruvo, Pr. Leclerc, Dr. Renaud Lopes, Dr. Romain Viard, Dr. Gregory Kuchcinski, Julien Dumont
– Neuropsychologists: Pr. Kathy Dujardin, M. Delliaux, M. Brion
– Neurosurgeons: Dr. Gustavo Touzet, Pr. Nicolas Reyns
– Neurophysiologists: Pr. Arnaud Delval
– Clinical Assistants: Valerie Santraine, Marie Pleuvret, Nolwen Dautrevaux, Victor Laugeais, Morgane Coeffet
– Clinical Trials Vigilance Unit: Thavarak Ouk, Camille Potey, Celine Leclercq, Elise Gers

## – Paris

– Neurologists: Jean‐Christophe Corvol, Marie‐Vidailhet, Elodie Hainque, Marie‐Laure Welter, Lucette Lacomblez, David Grabli, Emmanuel Roze, Yulia Worbe, Cécile Delorme, Hana You, Jonas Ihle, Raquel Guimeraes‐Costa, Florence Cormier‐Dequaire, Aurélie Méneret, Andréas Hartmann, Louise‐Laure Mariani
– Neuroradiologists: Stéphane Lehericy
– Neuropsychologists: Virginie Czernecki, Fanny Pineau, Frédérique Bozon, Camille Huiban, Eve Benchetrit
– Neurosurgeons: Carine Karachi, Soledad Navarro, Philippe Cornu
– Clinical Assistants: Arlette Welaratne, Carole Dongmo‐Kenfack
– Nurses: Lise Mantisi, Nathalie Jarry, Sophie Aix, Carine Lefort

## – Nantes

– Neurologists: Dr. Tiphaine Rouaud, Pr. Philippe Damier, Pr. Pascal Derkinderen, Dr. Anne‐Gaelle Corbille
– Neuroradiologists: Dr. Elisabeth Calvier‐Auffray
– Neuropsychologists: Laetitia Rocher, Anne‐Laure Deruet
– Neurosurgeons: Dr. Raoul Sylvie, Dr. Roualdes Vincent
– Clinical Assistants: Le Dily Séverine

## – Clermont‐Ferrand

– Neurologists: Dr. Ana Marques, Dr. Berangere Debilly, Pr. Franck Durif, Dr. Philippe Derost, Dr. Charlotte Beal
– Neuroradiologists: Carine Chassain
– Neuropsychologists: Laure Delaby, Tiphaine Vidal
– Neurosurgeons: Pr. jean Jeacques Lemaire
– Clinical Assistants: Isabelle Rieu, Elodie Durand

## – Marseille

– Neurologists: Pr. Alexandre Eusebio, Pr. Jean‐Philippe Azulay, Dr. Tatiana Witjas, Dr. Frédérique Fluchère, Dr. Stephan Grimaldi
– Neuroradiologists: Pr. Nadine Girard
– Neuropsychologists: Eve Benchetrit, Marie Delfini
– Neurosurgeons: Dr. Romain Carron, Pr. Jean Regis, Dr. Giorgio Spatola
– Clinical Assistants: Camille Magnaudet

## – Poitiers

– Neurologists: Dr. Ansquer Solène, Dr. Benatru Isabelle, Dr. Colin Olivier, Pr. Houeto JL
– Neuroradiologists: Pr. Guillevin Remy
– Neuropsychologists: Fradet Anne, Anziza Manssouri, Blondeau Sophie
– Neuropsychiatrist: Dr. Richard Philippe
– Neurosurgeons: Dr. Cam Philippe, Dr. Page Philippe, Pr. Bataille Benoit
– Clinical Assistants: Rabois Emilie, Guillemain Annie

## – Rennes

– Neurologists: Dr. Drapier Sophie, Dr. Frédérique Leh, Dr. Alexandre Bonnet, Pr. Marc Vérin
– Neuroradiologists: Dr. Jean‐Christophe Ferré
– Neuropsychologists: Mr Jean François Houvenaghel
– Neurosurgeons: Pr. Claire Haegelen
– Clinical Assistants: Francoise Kestens, Solenn Ory

## – Bordeaux

– Neurologists: Pr. Pierre Burbaud, Dr. Nathalie Damon‐Perriere, Pr. Wassilios Meissner, Pr. Francois Tison, Dr. Stéphanie Bannier, Dr. Elsa Krim, Pr. Dominique Guehl
– Neuroradiologists: Sandrine Molinier‐Blossier, Morgan Ollivier, Marion Lacoste
– Neuropsychologists: Nicolas Auzou, Marie Bonnet
– Neurosurgeons: Pr. Emmanuel Cuny, Dr. Julien Engelhardt
– Clinical Assistants: Olivier Branchard, Clotilde Huet, Julie Blanchard

## – Toulouse

– Neurologists: Pr. Rascol Olivier, Dr. Christine Brefel Courbon, Dr. Fabienne Ory Magne, Dr. Marion Simonetta Moreau
– Psychiatrists: Pr. Christophe Arbus
– Neuroradioligsts: Pr. Fabrice Bonneville et Dr. Jean Albert Lotterie
– Neuropsychologists: Marion Sarrail
– Neurosurgeons: Pr. Patrick Chaynes, Pr. François Caire
– Clinical Assistants: Estelle Harroch

## – Rouen

– Neurologists: Pr. David Maltete, Dr. Romain Lefaucheur, Dr. Damien Fetter
– Neuroradiologists: Dr. Nicolas Magne
– Neuropsychologists: Sandrine Bioux, Maud Loubeyre, Evangéline Bliaux, Dorothée Pouliquen
– Neurosurgeons: Pr. Stéphane Derrey
– Nurses: Linda Vernon
– Biologists: Dr. Frédéric Ziegler

## – Strasbourg

– Neurologists: Mathieu Anheim, Ouhaid Lagha‐Boukbiza, Christine Tranchant, Odile Gebus, Solveig Montaut
– Neuroradiologists: Stéphane Kremer
– Neuropsychologists: Nadine Longato, Clélie Phillips
– Neurosurgeons: Jimmy Voirin, Marie des Neiges Santin, Dominique Chaussemy
– Psychiatrists: Dr. Amaury Mengin

## – Nice

– Neurologists: Dr. Caroline Girodana, Dr. Claire Marsé
– Neuroradiologists: Lydiane Mondot
– Psychiatrists: Bruno Giordana, Robin Kardous
– Neuropsychologists: Bernadette Bailet, Héloise Joly
– Neurosurgeons: Denys Fontaine, Dr. Aurélie Leplus
– IDE: Amélie Faustini
– Clinical Assistants: Vanessa Ferrier

## – Amiens

– Neurologists: Pr. Pierre Krystkowiak, Dr. Mélissa Tir
– Neuroradiologists: Pr. Jean‐Marc Constans
– Neuropsychologists: Sandrine Wannepain
– Clinician Psychologists: Audrey Seling
– Neurosurgeons: Dr. Michel Lefranc
– Clinical Assistants: Stéphanie Blin
– Parkinson coordinator IDE: Béatrice Schuler

## – Lyon

– Neurologists: Pr. Stephane Thobois, Dr. Teodor Danaila, Dr. Chloe Laurencin
– Neuroradiologists: Pr. Yves Berthezene, Dr. Roxana Ameli
– Neuropsychologists: Helene Klinger
– Neurosurgeons: Dr. Gustavo Polo, Patrick Mertens
– Nurses: A Nunes
– Clinical Assistants: Elise Metereau

## – Nancy

– Neurologists: Dr. Lucie Hopes, Dr. Solène Frismand
– Neuroradiologists: Dr. Emmanuelle Schmitt
– Neuropsychologists: Mylène Meyer, Céline Dillier
– Neurosurgeons: Pr. Sophie Colnat
– Clinical Assistants: Anne Chatelain

## – Hospital Fondation Rothschild

– Neurologists: Dr. Jean‐ Philippe Brandel, Dr. Cécile Hubsch, Dr. Patte Karsenti, Dr. Marie Lebouteux, Dr. Marc Ziegler
– Neuroradiologists: Dr. Christine Delmaire, Dr. Julien Savatowky
– Neuropsychologists: Juliette Vrillac, Claire Nakache
– Neurosurgeons: Dr. Vincent D'Hardemare
– Clinical Assistants: Lhaouas Belamri

## – Hospital Foch

– Neurologists: Dr. Valérie Mesnage, Dr. Cécilia Bonnet, Dr. Jarbas Correa Lino Junior
– Neurophysiologist: Dr. Camille Decrocq
– Neuroradiologists: Dr. Anne Boulin
– Neuropsychologists: Elodie Dupuy, Inès Barre
– Psychiatrists: Dr. Bérénice Gardel
– Neurosurgeons: Pr. Béchir Jarraya
– Clinical Assistants: Delphine Lopez
– Coordinator: Catherine Ziz

## – CATI (MRI Acquisition Management, Preprocessing, and Data Management)

– David Gay, Robin Bonicel, Fouzia El Mountassir, Clara Fischer, Jean‐François Mangin, Marie Chupin, and Yann Cointepas.

## – CRB of Lille (Center of Biological Resources)

– Bertrand Accart, Patrick Gelé, Florine Fievet, Matthieu Chabel, Virginie Derenaucourt, Loïc Facon, Yanick Tchantchou Njosse, and Dominique Deplanque.

## – Data Management of Lille

– Alain Duhamel, Lynda Djemmane, Florence Duflot, and Hajar Chouiki.

### Peer Review

The peer review history for this article is available at https://publons.com/publon/10.1002/brb3.70101.

## Supporting information



Supporting Information

## Data Availability

The data that support the findings of this study are available from the corresponding author upon reasonable request.
