# Supplementary material for: Parkinson's Disease, Speech and Neurosurgery
Source: Brain Behav. 2025 Apr 29;15(5):e70101. doi: 10.1002/brb3.70101 (PMC12040730; doi:10.1002/brb3.70101)
Supplement: Supplementary file 1 — Supporting Information [file BRB3-15-e70101-s001.docx]

**SUPPLEMENTAL DATA**

**Supplemental Table 1.** **Characteristics of the study population**

| **Characteristic** |  |
| --- | --- |
| Vascular or infectious complication of surgery | 12 (3.0) |
| Hoehn and Yahr score at V0  OFF  ON | 2.5 (2; 3)  1 (1; 2) |
| CGIS at V0 | 4.5 (4; 5) |
| PDQ39: total score at  V0  V1 | 50.8 ± 19.8  43.3 ± 22.9 |
| Sum of MDS-UPDRS III axial scores at V0 OFF drug | 7.9 ± 4.7 |
| MDS-UPPDRS I at V0 | 10.4 ± 5.3 |
| MDS-UPDRS II  V0  V1 | 18.6 ± 7.6  6.3 ± 5.8 |
| MDS-UPDRS IV at V0 | 8.3 ± 3.8 |
| MDS-UPDRS III at V1  ON drug OFF stim  OFF drug ON stim  BEST ON  WORST OFF | 19.1 ± 12.0  22 ± 12.4  10.8 ± 7.2  44.5 ± 16.1 |
| MoCA at V0 | 27 (26; 29) |
| LARS-total at V0 | -29 (-32; -25) |
| HAM-A at V0 | 4 (2; 8) |
| FOGQ4 at V0  Pacing frequency (Hertz)  Pacing pulse width (μs) | 2 (0; 3)  130 ± 62.5  60 ± 28.4 |

Quantitative variables are shown as mean (± SD), or median (Q1; Q3). Categorical variables are expressed as n (%).

MDS-UPDRS: Movement Disorders Society-Unified Parkinson Disease Rating Scale; CGIS: clinical global impression of severity; PDQ39: Parkinson’s disease quality of life – 39; BEST ON: with both stimulation and drug; WORST OFF: without stimulation or drug; MoCA: Montreal cognitive assessment; LARS-total: Lille apathy rating scale; HAM-A: Hamilton anxiety scale; FOGQ4: question 4 of freezing of gait questionnaire.

**Supplemental Table 2.** **Results of univariate analysis of preoperative clinical predictive factors in the MDS-UPDRS III.1 1 year after STN-DBS**

| **Outcome and predictive variables** | **Evolution of scores at V1** | | | **p**  **value** |
| --- | --- | --- | --- | --- |
|  | **I**  **(N=56)** | **S**  **(N=152)** | **W**  **(N=101)** |  |
| **Sex: male**  **MDS-UPDRS III.1 ≤1/4**  **MDS-UPDRS II OFF ^1^**  **MDS-UPDRS III OFF**  **Left electrode intensity (V) ^2^**  **Disease duration (years) ^3^**  Age, V0  Surgery complications  PDQ39 total score V0  LEDD V0 **^4^**  Hoehn and Yarr OFF V0 **^5^**  MoCA V0 **^6^**  LARS total score V0 **^7^**  HAM-A V0 **^8^**  FOGQ4 V0 **^5^**  MDS-UPDRS I V0  MDS-UPDRS III ON V0 **^9^**  MDS-UPDRS IV V0  Sum of axial scores V0 **^10^** | 45 (80)  37 (66)  19.8 ± 8  44.9 ± 13  2 ± 1  12.3 ± 5  59.7 ± 7  1 ± 2  52.2 ± 17  1290 ± 532  2.5 (2.0, 3.0)  27 (26, 28)  -28 (-31, -24)  5 (3, 8)  3 (1, 3)  9.9 ± 5  12.3 ± 7  7.9 ± 4  9.0 ± 5 | 90 (59)  144 (95)  17.5 ± 8  39.6 ± 15  2 ± 1  11.6 ± 4  59.5 ± 8  3 ± 2  52.3 ± 19  1372 ± 535  2.5 (2.0, 3.0)  27 (25, 28)  -29 (-32, -26)  5 (3, 9)  2 (0, 3)  11.2 ± 5  10.0 ± 7  8.2 ± 4  7.3 ± 5 | 68 (67)  96 (95)  19.9 ± 7  42.3 ± 14  2.4 ± 1  10.3 ± 3  60.3 ± 8  5 ± 5  49.5 ± 22  1291 ± 539  2.5 (2.0, 3.0)  28 (26, 29)  -29 (-32, -25)  5 (2, 8)  2 (0, 3)  10.2 ± 5  10.3 ± 7  8.8 ± 4  7.8 ± 5 | **0.016**  **< 0.001**  **0.032**  **0.046**  **< 0.001**  **0.008**  0.71  0.45  0.52  0.43  0.78  0.069  0.58  0.44  0.11  0.15  0.11  0.29  0.062 |

Quantitative variables are expressed as mean (± SD), or median (Q1, Q3). Categorical variables are expressed as n (%). Significant results are shown in bold.

I/S/W: improvement/stable/worse; STN-DBS: sub-thalamic nucleus deep brain stimulation; FOGQ4: question 4 of the Freezing of Gait Questionnaire; HAM-A: Hamilton Anxiety Scale; LEDD: levodopa equivalent daily dose; LARS: Lille Apathy Rating Scale; MDS-UPDRS: Movement Disorders Society-Unified Parkinson Disease Rating Scale; MoCA: Montreal Cognitive Assessment; V0: preoperative; PDQ39: Parkinson’s disease quality of life question 39; V1: 1 year after surgery.

**^1^**Calculated for 369 patients. **^2^**Calculated for 209 patients. **^3^**Calculated for 308 patients.

**^4^**Calculated for 297 patients. **^5^**Calculated for 295 patients. **^6^**Calculated for 255 patients.

**^7^**Calculated for 297 patients. **^8^**Calculated for 296 patients. **^9^**Calculated for 305 patients.

**^10^**Calculated for 308 patients.

**Supplemental Table 3.** **Results of univariate analysis of preoperative clinical predictive factors of PDQ39-Q34 1 year after STN-DBS**

| **Outcome and predictive variables** | **Evolution mode of scores at V1** | | | **p value** |
| --- | --- | --- | --- | --- |
|  | **I**  **(N=82)** | **S**  **(N=160)** | **W**  **(N=159)** |  |
| Sex: male  Age (years), V0  Disease duration (years)  No. of surgery complications  Left electrode intensity (V)  **Preoperative dysarthria ≤1/4**  **PDQ39 total score V0**  LEDD V0  Hoehn and Yarr OFF V0  MoCA V0  LARS total score V0  **HAMA V0**  FOGQ4 V0  MDS-UPDRS I V0  MDS-UPDRS II OFF V0  MDS-UPDRS III OFF V0  MDS-UPDRS III ON V0  Sum of axial scores V0  MDS-UPDRS IV V0 | 49 (60)  60 ± 7  12 ± 5  2 ± 3  2 ± 1  19 (23)  56 ± 19  1338 ± 645  2.5 (2, 3)  27 (25, 29)  -28 (-31, -25)  5 (3, 9)  2 (0, 3)  11 ± 6  19 ± 7  42 ± 14  11 ± 7  8 ± 5  9 ± 4 | 110 (69)  61 ± 7  11 ± 4  3 ± 2  2 ± 1  77 (48)  49 ± 19  1310 ± 504  2.5 (2, 3)  27 (25, 29)  -29 (-32, -24  4 (2, 7)  2 (0, 3)  10 ± 5  18 ± 8  42 ± 14  11 ± 7  7 ± 4  8 ± 4 | 104 (65)  60 ± 8  11 ± 4  7 ± 5  2 ± 1  115 (72)  51 ± 20  1380 ± 590  2.5 (2, 3)  28 (26, 29)  -29 (-32, -26)  5 (3, 9)  2 (0, 3)  10 ± 5  19 ± 8  42 ± 17  11 ± 8  8 ± 5  8 ± 4 | 0.38  0.46  0.21  0.41  0.45  **< 0.001**  **0.015**  0.56  0.86  0.65  0.23  **0.049**  0.71  0.96  0.50  0.98  0.88  0.31  0.75 |

Quantitative variables are expressed as mean (± SD), or median (Q1, Q3). Categorical variables are shown as n (%). Significant results are shown in bold.

I/S/W: improvement/stability/worsening; FOGQ4: question 4 of the freezing of gait questionnaire; HAMA: Hamilton anxiety scale; LEDD: levodopa equivalent daily dose; LARS: Lille apathy rating scale; MDS-UPDRS: Movement Disorders Society-Unified Parkinson Disease Rating Scale; MoCA: Montreal cognitive assessment; V0: preoperative; PDQ39: Parkinson’s disease quality of life 39 questions; V1: 1-year post-surgery.

**Supplemental Table 4.** **Results of univariate analysis of preoperative clinical predictive factors of PDQ39-Q35 1 year after STN-DBS**

| **Outcome and predictive variables** | **Evolution mode of scores at V1** | | | **p value** |
| --- | --- | --- | --- | --- |
|  | **I**  **(N=96)** | **S**  **(N=178)** | **W**  **(N= 132)** |  |
| Sex: male  Age (years), V0  Disease duration (years)  No. of surgery complications  Left electrode intensity (V)  **PDQ39-Q34 ≤1/4**  **PDQ39 total score V0**  LEDD V0  Hoehn and Yarr OFF V0  MoCA V0  LARS total score V0  HAMA V0  FOGQ4 V0  MDS-UPDRS I V0  **MDS-UPDRS II OFF V0**  MDS-UPDRS III OFF V0  MDS-UPDRS III ON V0  Sum of axial scores V0  MDS-UPDRS IV V0 | 68 (71)  60 ± 7  11 ± 4  2 ± 2  2 ± 1  33 (34)  53 ± 17  1297 ± 512  2.5 (2, 3)  27 (25, 29)  -29 (-32, -26)  4 (3, 8)  2 (0, 3)  10 ± 5  18 ± 7  41 ± 14  10 ± 7  8 ± 5  8 ± 4 | 112 (63)  61 ± 7  11 ± 4  4 ± 2  2 ± 1  140 (79)  48 ± 20  1379 ± 518  2.5 (2, 3)  27 (25, 28)  -29 (-32, -24)  4 (2, 8)  2 (0, 3)  10 ± 5  18 ± 7  43 ± 15  11 ± 7  8 ± 5  8 ± 4 | 86 (65)  60 ± 9  11 ± 4  6 ± 5  2 ± 1  107 (81)  54 ± 19  1332 ± 660  2.5 (2, 3)  28 (26, 29)  -29 (-32, -25)  5 (3, 9)  2, (0, 3)  11 ± 5  20 ± 8  42 ± 16  11 ± 7  8 ± 5  9 ± 4) | 0.42  0.33  0.95  0.42  0.79  **<0.001**  **0.031**  0.46  0.39  0.35  0.70  0.21  0.93  0.48  **0.009**  0.70  0.88  0.72  0.63 |

Quantitative variables are expressed as mean (SD), or median (Q1, Q3). Categorical variables are shown as n (%). Significant results are shown in bold.

I/S/W: improvement/stability/worsening; FOGQ4: question 4 of the freezing of gait questionnaire; HAMA: Hamilton anxiety scale; LEDD: levodopa equivalent daily dose; LARS: Lille apathy rating scale; MDS-UPDRS: Movement Disorders Society-Unified Parkinson Disease Rating Scale; MoCA: Montreal cognitive assessment; V0: preoperative; PDQ39: Parkinson’s disease quality of life 39 questions; V1: 1 year post-surgery.

**Supplemental Table 5. Independent predictive factors retained in the final predictive model of PDQ39-Q34 score change at V1**

|  | **Improvement vs. stability** | | **Worsening vs. stability** | | **Overall**  **p value** |
| --- | --- | --- | --- | --- | --- |
|  | **OR [95%CI]** | **p value** | **OR [95%CI]** | **p value** |  |
| PDQ39-Q34 V0 >1 | 3.09 [1.70‒5.61] | 0.0002 | 0.36 [0.22‒0.57] | <0.0001 | <0.001 |

The median (Q1, Q3) value of the polytomous discrimination index (PDI) for the final predictive models was 0.456 (0.449‒0.459).

PDQ39: Parkinson’s disease quality of life; V0: preoperative; V1: 1-year post-surgery; OR: Odds ratio; CI: confidence interval.

**Supplemental Table 6. Independent predictive factors retained in the final predictive model of PDQ39-Q35 score change at V1.**

|  | **Improvement vs. stability** | | **Worsening vs. stability** | | **Overall**  **p value** |
| --- | --- | --- | --- | --- | --- |
|  | **OR [95%CI]** | **p-value** | **OR [95%CI]** | **p-value** |  |
| PDQ39-Q35 V0 >1 | 7.88 [4.44‒14.00] | <0.0001 | 0.71 [0.40‒1.36] | 0.24 | <0.001 |
| MDS-UPDRS II OFF V0 | 0.97 [0.93‒1.01] | 0.1244 | 1.04 [1.01‒1.08] | 0.009 | <0.001 |

The median (Q1, Q3) value of the polytomous discrimination index (PDI) for the final predictive models was 0.545 (0.538‒0.554).

MDS-UPDRS: Movement Disorders Society-Unified Parkinson Disease Rating Scale; PDQ39: Parkinson’s disease quality of life; V0: preoperative; V1: 1-year post-surgery; OR: Odds ratio; CI: confidence interval.
